# Medical Education

**Published:** 1868-03

**Authors:** 


Medical Education.—E. D. Mansfield, Esq., the very intelligent special contributor of the *Cincinnati Gazette*, has the following remarks in a recent number of that paper on Medical Education. There are thoughts in the extract that are well worthy the attention of all who have anything to do with educating young men for the medical profession.

" Dr. McDermott, Surgeon-General of Ohio, says :

" None but graduates of regular medical schools were admitted to examination, and yet over eighty per cent. of these were rejected for incompetence. The ignorance betrayed by many of the candidates was deplorable, proving that the diploma of a medical college has ceased to be of any value as evidence of capacity.

"If it was the duty of the State, as all concede, to provide competent physicians for the soldiers, it is no less a duty to make similar provision for the citizens; and yet those rejected candidates, with hundreds of others equally incompetent, are now scattered over the State, pursuing their fatal trade with criminal recklessness."—*Dayton Journal.*

"Dr. McDermott deserves thanks for this brief exposition of what intelligent men have long observed,—that medical education is fearfully neglected. Of course, there is another side to this. The well educated, practicing physicians were not among those examined, and make a large class. No one supposes that the medical profession has not many men of skill, science and high character. But there is no profession which has within it so large a proportion of unintelligent, unthinking, uneducated members. How happens this? Simply because the standards of medical education, the medical colleges, give men diplomas for a minimum of medical knowledge, and no knowledge of anything else. They go into the community with that diploma to try experiments of all sorts on the bodies and minds of their fellow citizens, with no judge, as with lawyers, to decide cases; no presbyteries or bishops, like the clergy, to determine their merits, and not even a chamber of commerce, like the merchants, to settle character. They go out with lancet and pill box, with calomel and quinine, to deal with the *patient* community. Few escape so well as one who, being ordered to take pills, left the box untouched on the mantel piece. The doctor came in the morning:

> Quoth the Doctor—"My medicine did good,"
> "It did no harm, for yonder it hath stood."

"It is absurd to suppose that young men, without any preliminary education, can be fitted for the medical profession by hearing four lectures a day for six months, with a few evenings in an anatomical room. The result is very injurious to the profession itself; for if the people must employ doctors haphazard, without any peculiar learning, most of them will be as likely to employ the disciple of Hahneman, *similis similibus;* or, the botanic number six; or the hydropathist with ten gallons of extra water; or the galvanist with four metal rings, as the doctor of the regular profession."

